# The *In Vitro* and *In Vivo* Antitumor Effects of Clotrimazole on Oral Squamous Cell Carcinoma

**DOI:** 10.1371/journal.pone.0098885

**Published:** 2014-06-03

**Authors:** Juan Wang, Lihua Jia, Zirong Kuang, Tong Wu, Yun Hong, Xiaobing Chen, W. Keung Leung, Juan Xia, Bin Cheng

**Affiliations:** 1 Department of Oral Medicine, Guanghua School of Stomatology, Guangdong Provincial Key Laboratory of Stomatology, Sun Yat-sen University, Guangzhou, Guangdong, China; 2 Oral Diagnosis and Polyclinics, Prince Philip Dental Hospital, Faculty of Dentistry, The University of Hong Kong, Hong Kong SAR, China; Winship Cancer Institute of Emory University, United States of America

## Abstract

**Background:**

Clotrimazole is an antifungal imidazole derivative showing anti- neoplastic effect in some tumors, but its anticancer potential is still unclear in oral squamous cell carcinoma (OSCC). The aim of this study was to evaluate the antitumor effect of clotrimazole, and to investigate the possible mechanism of clotrimazole-mediated antitumor activity in OSCC.

**Methodology:**

In vitro experiments, the cell viability and clonogenic ability of three human OSCC cell lines CAL27, SCC25 and UM1 were detected after clotrimazole treatment by CCK8 assay and colony formation assay. Cell cycle progression and apoptosis were assessed by flow cytometry, and the involvement of several mediators of apoptosis was examined by western blot analysis. Then, the in vivo antitumor effect of clotrimazole was investigated in CAL27 xenograft model. Immunohistochemistry and western blot analysis were performed to determine the presence of apoptotic cells and the expression of Bcl-2 and Bax in tumors from mice treated with or without clotrimazole.

**Results:**

Clotrimazole inhibited proliferation in all three OSCC cell lines in a dose-and time-dependent manner, and significantly reduced the colony formation of OSCC cells in vitro. Clotrimazole caused cell cycle arrest at the G_0_/G_1_ phase. In addition, clotrimazole induced apoptosis in OSCC cells, and significantly down-regulated the anti-apoptotic protein Bcl-2 and up-regulated the pro-apoptotic protein Bax. Notably, clotrimazole treatment inhibited OSCC tumor growth and cell proliferation in CAL27 xenograft model. Clotrimazole also markedly reduced Bcl-2 expression and increased the protein level of Bax in tumor tissues of xenograft model.

**Conclusion:**

Our findings demonstrated a potent anticancer effect of clotrimazole by inducing cell cycle arrest and cellular apoptosis in OSCC.

## Introduction

Clotrimazole is an antifungal imidazole derivative which has been used in clinic for more than 20 years. Since the early 1980s, clotrimazole has been available for the treatment of oral candidiasis, skin infections, and for prophylaxis of oropharyngeal candidiasis in immunocompromised patients [1], [2]. In addition to its antifungal properties, a few studies have shown its anticancer properties. Clotrimazole had growth inhibition effects on several human cancer cell lines, such as lung carcinoma, colorectal cancer, breast cancer and endometrial cancer [3]–[6]. Clotrimazole also inhibited tumor growth in xenograft rat model of intracranial gliomas (C6 and 9L) and prolonged the rat survival [7].

Previous study showed that clotrimazole, as calmodulin antagonist, inhibited the proliferation of human cancer cells via disrupting cellular Ca^2+^ homeostasis. It released Ca^2+^ from intracellular stores while inhibiting Ca^2+^ influx and blocking IK channels [8], [9]. Further studies demonstrated that clotrimazole blocked cell cycle in G_1_ phrase and induced apoptosis [10]–[12]. Moreover, clotrimazole effectively decreased glucose consumption and energy metabolism by inhibiting glycolysis and ATP production, and then led to reduction of tumor cell viability [13], [14]. However, these previous studies about the anticancer effects and mechanisms of clotrimazole were mostly involved in adenocarcinomas. The effects of clotrimazole on squamous cell carcinoma (SCC) remain relatively unknown. Squamous cell carcinoma and adenocarcinoma are different histological types, which is one of the most important reasons for the different patient responses to the same anticancer treatment [15]. Thus, it is necessary and interesting to investigate the effects of clotrimazole on squamous cell carcinoma, such as oral squamous cell carcinoma (OSCC).

OSCC is the most common oral malignancy, representing up to 80–90% of all malignant neoplasms of the oral cavity [16]. The 5-year survival rate of OSCC patients has not improved significantly in the last several decades [17]. Therefore, it is necessary to identify novel and effective therapeutic agents for the treatment of OSCC. Whether clotrimazole directly affects the proliferation of OSCC cells has not been reported. Thus, the present study was designed to demonstrate the antitumor effects of clotrimazole on OSCC cells and to investigate the possible underlying mechanisms. We observed that clotrimazole significantly inhibited OSCC cell proliferation both in vitro and in vivo. Moreover, clotrimazole induced cell apoptosis and led to a significant down-regulation of anti-apoptotic protein Bcl-2 and up-regulation of pro-apoptotic protein Bax.

## Materials and Methods

### Cell lines and Materials

Three human oral squmous cell carcinoma cell lines (CAL27, SCC25 and UM1) were included in this study. CAL27 (ATCC number: CRL-2095) and SCC25 (ATCC number: CRL-1628) were purchased from the American Type Culture Collection (ATCC, Manassas, VA, USA). UM1 cell line was a gift from Professor Hongzhang Huang (Department of Oral and Maxillofacial Surgery, Guanghua School of Stomatology, Sun Yat-sen University, China). SCC25 and UM1 cells were cultured in 1∶1 mix of Dulbecco's Modified Eagle Medium and Ham F12 medium (DMEM/F12) (Invitrogen, Carlsbad, CA, USA) supplemented with penicillin (100 units/ml), streptomycin (100 µg/ml) and 10% (v/v) fetal bovine serum (FBS) (GIBCO, Grand Island, NY, USA). CAL27 cells were cultured in DMEM supplemented with the same concentrations of FBS and penicillin and streptomycin. Cells were incubated at 37°C in a humidified atmosphere of 5% CO_2_.

Clotrimazole was purchased from Sigma Chemical (St. Louis, MO, USA) and dissolved in dimethyl sulfoxide (DMSO) (Sigma Chemical). The concentration of DMSO was kept under 0.1% throughout all the experiments to avoid cytotoxicity. Antibodies against Bcl2, Bax, cleave-caspase3 and alpha-tublin were purchased from Cell Signaling Technology (Beverly, MA, USA). PCNA antibody was purchased from Fuzhou maxim (Fuzhou, China).

### CCK8 cell viability assay

Cell viability was assessed by a Cell counting Kit-8 (CCK8) assay (Dojindo, Kumamoto, Japan). OSCC cells (5×10^3^ cells/well) were plated into 96-well plates. After 24 h, the cells were treated with different concentrations of clotrimazole (0–80 µM) or DMSO (0.1%). After incubation with clotrimazole for 24 h, 48 h or 72 h, the CCK8 reagent was added to each well and cells were incubated for 2 h at 37°C. The absorbance (optical density) at 450 nm was measured.

### Cell colony formation assay

OSCC cells were seeded into 6-well plates in triplicate at a density of 1000 cells/well in 2 ml of medium containing 10% FBS. After 24 h, cultured cells were replaced with fresh culture medium containing DMSO or various concentrations of clotrimazole (10, 20 or 30 µM) at 37°C and 5% CO2. Cells were grown for 14 days. The culture medium was changed once every other day. The cell colonies were stained for 20 minutes with a solution containing 0.5% crystal violet and 25% methanol, followed by three rinses with tap water to remove excess dye. Colonies consisting of >50 cells were counted under a microscopy. All experiments were repeated in triplicate and the average values are presented.

### Cell cycle analysis

OSCC cells (2×10^5^ cells/well) were seeded in 6-well plates. After starvation with basal medium for 24 h, cells were treated with DMSO or clotrimazole (30 and 40 µM) for 24 h and then harvested by trypsinization. The cells were fixed with cold 70% ethanol and stained for total DNA content with RNase A and propidium iodide staining buffer (BD, San Diego, CA, USA) according to the manufacturer's instructions. A minimum of 10,000 cells were acquired per sample and cell cycle distribution was analyzed using a flow cytometer (Becton Dickinson, San Jose, CA, USA) and ModFit software V3.0 (Verity Software House, Topsham, ME, USA).

### Apoptosis analysis

Cell apoptosis was assessed using the Annexin V-FITC/Propidium Iodide (PI) double-staining apoptosis detection kit (KeyGen, China). OSCC cells were treated with DMSO or clotrimazole (30 and 40 µM) for 24 h. The cells were collected and stained according to the manufacturer's instructions. The apoptosis data acquisition and analysis was performed by a FACS Calibur flow cytometer. Basal apoptosis were identically determined on control cells.

### Western blot analysis

OSCC cells (2×10^5^ cells/well) were seeded in 6-well plates. After 24 h, the medium was replaced with fresh culture medium containing 40 µM clotrimazole or DMSO for 12, 24 and 48 h. Then cultured cells were lysed in RIPA buffer supplemented with protease and phosphatase inhibitors (Pierce, Rockford, IL, USA). The protein concentrations were measured using a BCA protein assay kit (Pierce, Rockford, IL, USA). Samples (40 µg/lane) were incubated at 100°C for 5 min, separated on 12% (w/v) SDS-PAGE gels, and electrophoretically transferred to a PVDF membrane (Millipore, Billerica, Massachusetts, USA). The blotted membrane was blocked with 5% non-fat milk for 2 h at room temperature. The membrane was incubated with primary antibodies against alpha-tublin, Bcl-2 and Bax overnight at 4°C, and then incubated with goat anti-mouse secondary antibody or goat anti-rabbit secondary antibody. The immunoreactive bands were detected using an enhanced chemiluminescence(ECL)detection system (Pierce, Rockford, IL, USA). Quantification of bands was performed using the gel analysis submenu of Image J software.

### Xenograft model analysis

Animal experiments were carried out in strict accordance with the recommendations in the guide for the care and use of laboratory animals. The protocol was approved by the Committee on the Ethics of Animal Experiments of the Sun Yat-sen University, China (Permit Number: 00054610). All surgery was performed under chloral hydrate anesthesia, and all efforts were made to minimize suffering. We purchased twenty-four female BALB/c nude mice (4 weeks old; 14–16 g) from the Animal Care Unit of Guangdong. The animals were bred in the Animal Care Unit of Sun Yet-sun University (Guangzhou, China) which provides specific pathogen-free conditions. Each mouse was inoculated with CAL27 cells (5×10^6^ cells per animal) subcutaneously into the back next to the right front limb. Ten days later, the xenografts were identifiable as a mass of more than 6 mm in maximal diameter in all recipients. Then mice were randomly assigned into control and treated groups (n = 12/group). The clotrimazole-treated group was injected 6 times a week intraperitoneally (i.p.) at 150 mg/kg/body per day for two weeks, whereas the control group received peanut oil (200 µl as vehicle). During this period, all the mice were examined every day to assess their health and any evidence of drug toxicity. Tumors were measured every two days with a standard caliper and tumor volumes were calculated as follows: tumor volume (mm^3^)  =  [tumor length (mm) ×tumor width (mm) ^2^]/2. Body weight of the mice was also recorded. At the end of the experiments (following treatment), the animals were anesthetized. Tumors were weighed after being separated from the surrounding muscles and dermis. For each group, tumor tissues were collected and separated into two parts. One was fixed with 10% neutral formalin and embedded in paraffin for immunohistochemistry examination, and the other aliquot were homogenized into tumor lysis buffer for western blot analysis.

### Immunohistochemistry

Immunohistochemistry was performed on xenograft tumor tissues. Antigen retrieval was performed by heating these tissue sections in 10 mmol/L citric acid buffer (pH 6.0) for 20 min. Tissue sections were blocked in 5% normal goat serum for 30 min, and incubated in 3% hydrogen peroxide to suppress endogenous peroxidase activity. Sections were then incubated with PCNA antibody (1∶100) and cleaved caspase-3 antibody (1∶1000) at 4°C overnight, followed by peroxidase-conjugated goat anti-rabbit secondary antibody for 1 h at room temperature. Finally, slides were treated with chromogen diaminobenzidine (DAB) (Dako, Carpinteria, CA, USA) for antigen detection and counterstained with hematoxylin. All sections were examined under light microscopy at ×200 magnification. For each section examined, cells in five randomly selected fields were counted. PCNA labeling index and cleaved caspase-3 labeling index was calculated as the percentage of positive cells over total number of cells examined.

### Statistical analysis

The data were presented as means ± standard deviation of at least three independent experiments. Statistical analysis of the results was performed using a two-tailed Student's t-test or one-way ANOVA and post hoc multiple comparison test with SPSS 16.0 software (IBM, Armonk, New York, USA). *P* value<0.05 were considered statistically significant.

## Results

### Clotrimazole inhibits human OSCC cells viability and reduces colony formation *in vitro*

**Figure 1:**
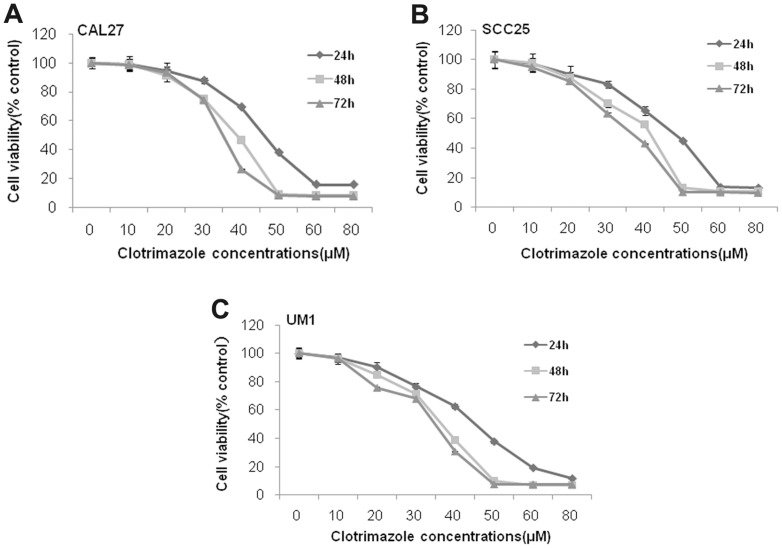
Clotrimazole inhibits OSCC cells proliferation. OSCC cells (CAL27, SCC25, and UM1) were treated with clotrimazole (0–80 µM) for 24 h, 48 h and 72 h, and cell viability was detected using a Cell Counting Kit-8 assay. The results presented as mean ± standard deviation values for three independent experiments.

**Figure 2:**
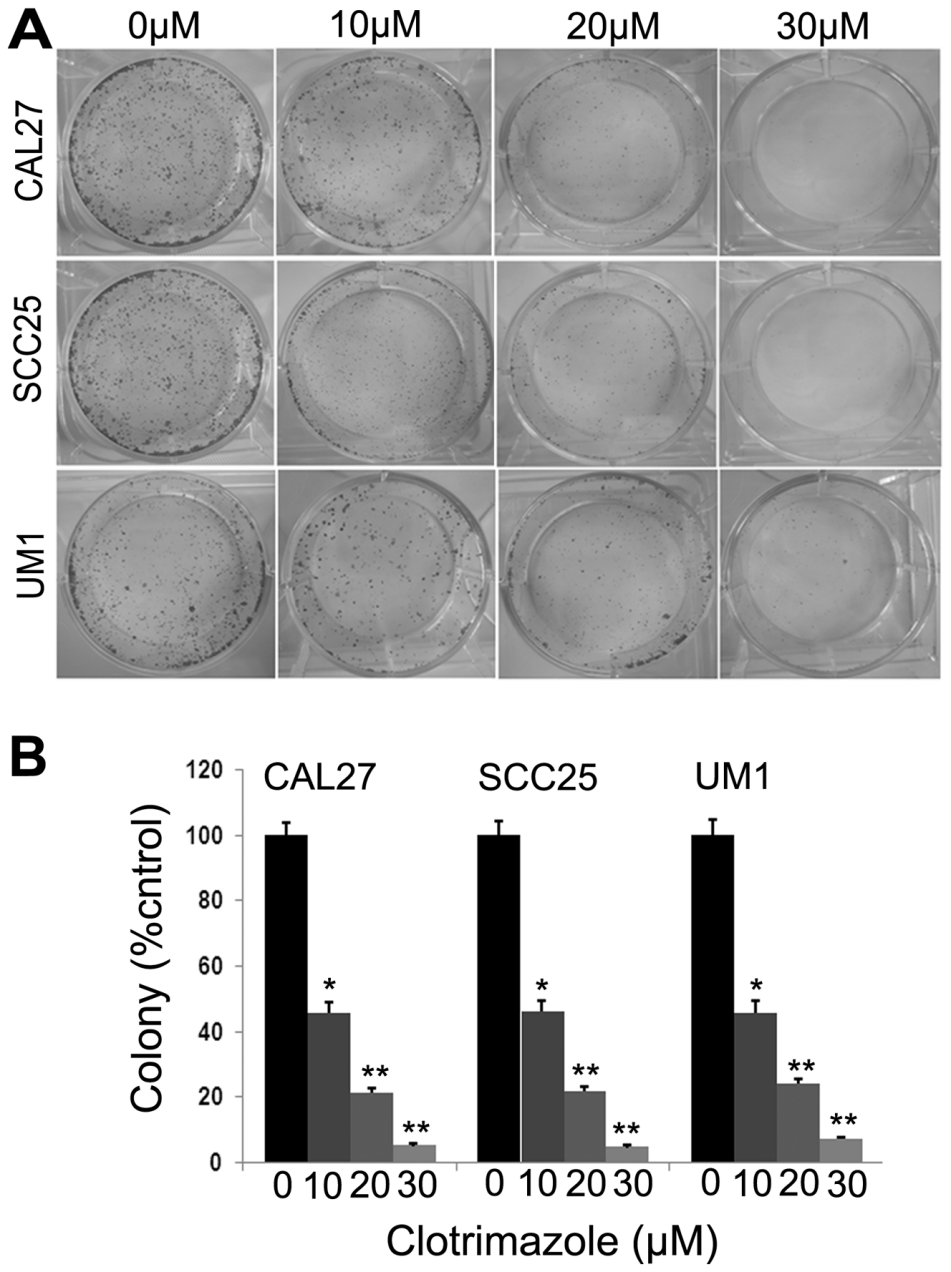
Clotrimazole inhibits colony formation of OSCC cells. OSCC cells (CAL27, SCC25, and UM1) grown in 6-well plates (1000 cells/well) were incubated with various concentrations of clotrimazole (0, 10, 20 and 30 µM) for two weeks. Cell colonies were stained and counted as described in the Methods section. The results presented as mean ± standard deviation values for three independent experiments. *P<0.05; **P<0.01 compared with solvent control.

To investigate the effects of clotrimazole on human OSCC cells growth in vitro, three OSCC cell lines CAL27, SCC25 and UM1 were used in our study. Treatment with clotrimazole led to significant inhibition of the cell viability in a dose-and time-dependent manner at each concentration (20, 30, 40 and 50 µM) compared with control cells (*P<*0.05, respectively) in these OSCC cell lines (Fig.1). After 40 µM clotrimazole treatment for 48 h, the inhibition rate reached 53.1% in CAL27 cells, 43.8% in SCC25 cells, and 61.5% in UM1 cells. The IC_50_ values for 48 h of clotrimazole treatment were 35.9 µM, 35.6 µM, and 31.4 µM in CAL27, SCC25, and UM1 cells, respectively. Therefore, we used 30 µM and 40 µM clotrimazole that encompassed concentrations above and below IC_50_ values for further experiments. Next, the ability of these three cell lines to form colonies with or without clotrimazole treatment was examined for a period of two weeks. Clotrimazole markedly decreased cell colony formation in a dose-dependent manner (Fig. 2A). At the low concentration of 10 µM, clotrimazole inhibited cell colony formation of CAL27, SCC25 and UM1 cells by 54.2%, 54.1% and 54.4%, respectively (*P*<0.05). Additionally, at the concentration of 30 µM clotrimazole, cell colony formation was strongly reduced by 95.0%, 95.5% and 93.0%, respectively (*P*<0.001), as compared with the respective control groups (Fig.2B). Collectively, our results indicated that clotrimazole inhibited the growth of OSCC cells in vitro.

### Clotrimazole induces OSCC cells cycle arrest

**Figure 3:**
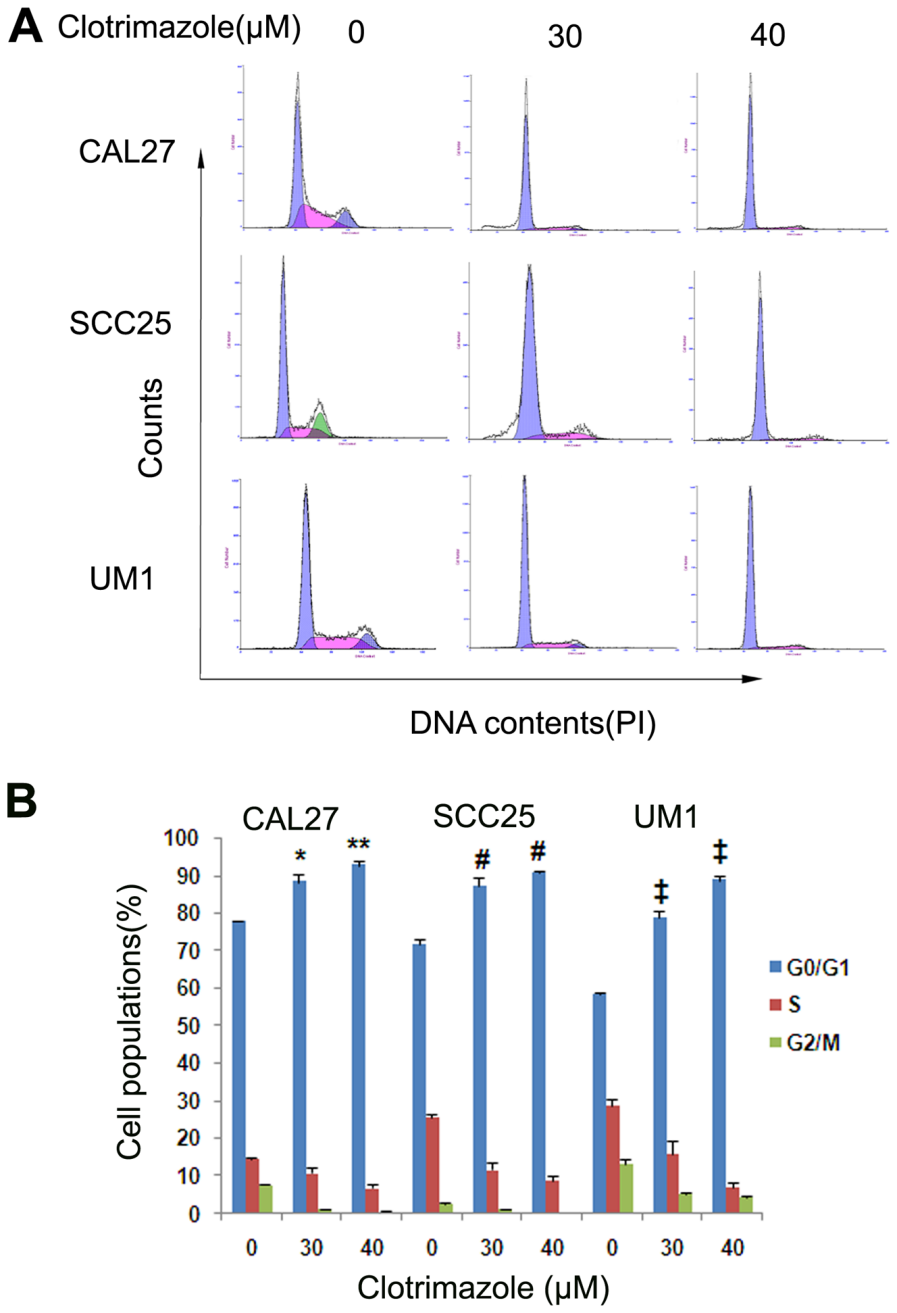
Clotrimazole induces G_0_/G_1_ cell cycle arrest in OSCC cells. OSCC cells were exposed to various concentrations of clotrimazole (0, 30 and 40 µM) for 24 h. Cell cycle distributions were analyzed by flow cytometry with PI staining. *P<0.05, **P<0.01 as compared with the CAL27 control cells; # *P*<0.01 as compared with the SCC25 control cells; ‡*P*<0.01 as compared with the UM1 control cells.

The possible effect of clotrimazole on cell cycle progression in OSCC cells was assessed by flow cytometry. Cells were treated with clotrimazole (0, 30 and 40 µM) for 24 h. Clotrimazole induced cell cycle arrest in a dose-dependent manner (Fig.3, *P*<0.05). Clotrimazole (40 µM) treatment significantly increased the proportion of OSCC cells in the G_0_/G_1_ phase, compared to the respective control cells (92.7±1.3% vs. 77.8±0.3% in CAL27, *P* = 0.002; 90.9±0.5% vs. 71.7±1.3% in SCC25, *P*<0.001; 87.4±0.4% vs.58.4±0.3% in UM1, *P*<0.001, respectively). Clotrimazole (40 µM) treatment also decreased the percentage of cells in the S phase of OSCC cell lines compared to the respective control cells (6.8±1.3% vs. 14.6±0.2% in CAL27, *P* = 0.023; 8.8±1.1% vs. 25.8±0.9% in SCC25, *P*<0.001; 7.1±1.1% vs.28.7±1.6% in UM1, *P* = 0.046, respectively).

### Clotrimazole induces apoptosis of OSCC cells

**Figure 4:**
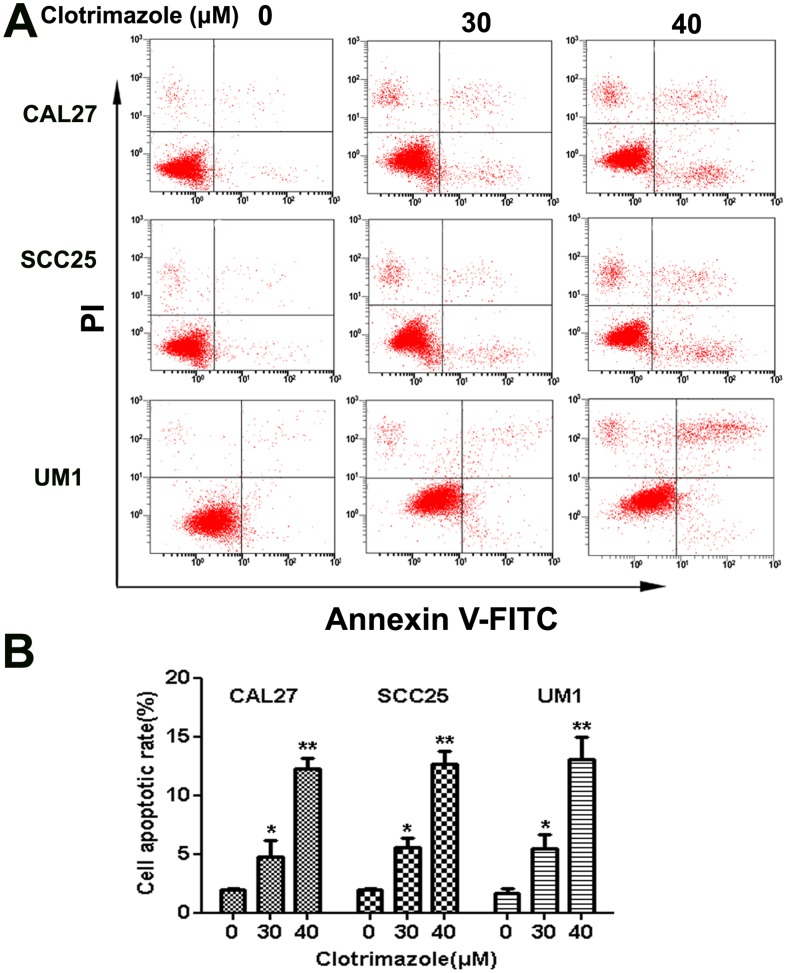
Clotrimazole induces apoptosis of OSCC cells. CAL27, SCC25 and UM1 cells were incubated with various concentrations of clotrimazole (0, 30 and 40 µM) for 24 h and labeled with Annexin V and propidium iodide (PI). (A) Apoptosis of OSCC cells was analyzed by flow cytometry. The bottom right quadrant represents the percentage of early apoptotic cells (Annexin V^+^/PI^−^), whereas the top right quadrant is the percentage of late apoptotic cells (Annexin V^+^/PI^+^). (B) Percentages of cells in apoptosis at each clotrimazole concentration. The results are presented as the mean of three similar experiments. *P<0.05; **P<0.01 compared with solvent control.

**Figure 5:**
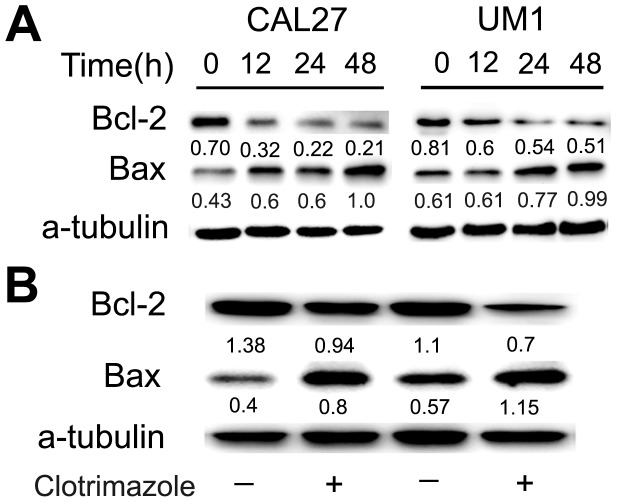
Clotrimazole regulates apoptotic protein levels in OSCC cells and tumor tissue. (A) CAL27 and UM1 cells were treated with 40 µM clotrimazole or DMSO for 12 h, 24 h, and 48 h. The expression of the anti-apoptotic protein Bcl-2 and the pro-apoptotic protein Bax was assessed by western blot. (B) The apoptosis-related protein expressions in tumors from control mice and clotrimazole-treated mice (150 mg/kg) were also analyzed by western blot analysis. Clotrimazole treatment decreased the expression of Bcl-2 and increased the level of Bax in clotrimazole-treated tumors compared with control animals. Data shows the representative of three independent experiments. Quantification of bands was performed using Image J software and the relative ratio was calculated by the density.

To further investigate whether clotrimazole initiates cellular apoptosis, OSCC cells were incubated with clotrimazole (0, 30, and 40 µM) for 24 h and analyzed by flow cytometry. The percentages of OSCC cells undergoing early apoptosis and late apoptosis were increased by clotrimazole treatment in a concentration-dependent manner (*P*<0.05) (Fig.4). The percentage of cells undergoing apoptosis was determined by the sum of cells in early and late apoptosis. Clotrimazole (40 µM) induced a significant increase in the proportion of apoptotic cells in CAL27, SCC25 and UM1 cells (12.3±0.9% vs. 2.0±0.1%, 12.6±1.1% vs. 1.9±0.1%, and 13.0±1.9% vs. 1.7±0.3%, respectively). The mechanisms underlying the apoptosis-inducing effect of clotrimazole in OSCC cells were assessed by western blot analysis. The levels of apoptosis-related proteins such as anti-apoptotic protein Bcl-2 and pro-apoptotic protein Bax were examined in total protein from tumor cells treated with DMSO or clotrimazole(40 µM) for 12 h, 24 h and 48 h. Our results showed that treatment with clotrimazole for 48 h induced a 3.3-fold and 1.6-fold decrease of Bcl2 protein expression in CAL27 and UM1 cells, respectively, as compared with respective control cells. Conversely, clotrimazole treatment induced a 2.3-fold and 1.6-fold increase of Bax protein expression in CAL27 and UM1 cells, respectively (Fig.5). These results indicate that an apoptotic mechanism is implicated in the clotrimazole-induced growth-inhibitory effects in OSCC cells.

### Clotrimazole inhibits OSCC tumor growth *in vivo*

**Figure 6:**
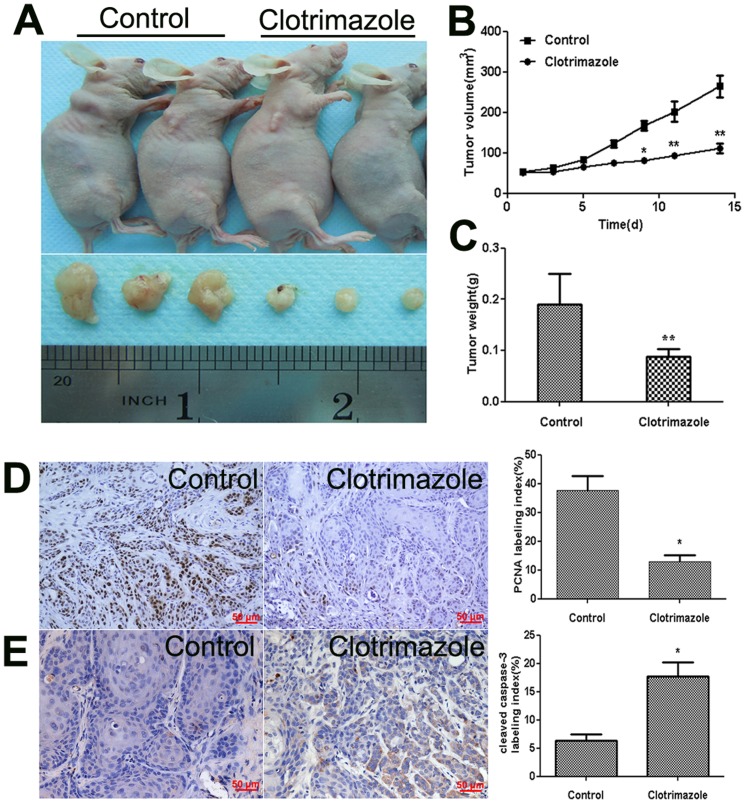
Clotrimazole slows the growth of human OSCC xenograft tumors in nude mice. A total of 5×10^6^ CAL27 cells/mouse were injected subcutaneously into the back next to the right front limb. When a tumor became palpable, clotrimazole (150 mg/kg/body) was administered intraperitoneally for 2 weeks, 6 times per week, control mice treated with equal volume of peanut oil. (A) Representative photographs of the gross tumors from nude mice treated with clotrimazole or peanut oil. (B) Graphs represent the average tumor volumes of CAL27 xenografts in mice from the control and clotrimazole-treated groups. (C) Graphs represent the average weight of tumors from the control and clotrimazole-treated groups. (D) PCNA expression in tumor tissues was assessed by IHC. The bar graph shows PCNA labeling index (PCI) in twelve tumors per each experimental group. PCI (%)  = positive tumor cells/total tumor cells×100%. (E) Cleaved caspase-3 expression in tumor tissues was assessed by IHC. The bar graph shows cleaved caspase-3 labeling index (CI) in twelve tumors per each experimental group. CI (%)  =  positive tumor cells/total tumor cells ×100%. *P<0.05; ** *P*<0.01 compared with control nude mice.

To evaluate whether clotrimazole affects tumor growth in vivo, the CAL27 cell line was selected for the establishment of the OSCC xenograft nude mouse model. Ten days later, the mice were randomly assigned into control and treated groups. The tumor volume (mean±SD) was 52.7±2.1 mm^3^ in control group and 51.7±2.3 mm^3^ in clotrimazole-treated group. Then clotrimazole was injected intraperitoneally at a dose of 150 mg/kg/day 6 days a week for two weeks. During the 14 days of drug or vehicle treatments, all the mice appeared to be healthy and there was no obvious sign or symptom of drug toxicity. The animal weight was not significantly different between the groups at any time point (Fig. S1). There are no obvious abnormalities were detected upon gross observation of the heart, liver, spleen, kidney, and gastrointestinal tract in clotrimazole-treated animals (Fig. S2). Consistent with our in vitro results, intraperitoneal administration of clotrimazole strikingly decreased the tumor volume of CAL27 cell xenograft in nude mice by 57.9% (*P* = 0.042, Fig.6A,B), and the mean weights of the excised tumors were approximately 53.6% lower in clotrimazole-treated mice than in control mice (*P* = 0.035, Fig.6C). The proliferation rates of tumor cell were assessed in situ by PCNA antigen labeling index. Our findings showed that clotrimazole treatment decreased tumor cell proliferation (Fig.6D).

### Clotrimazole induces apoptosis in OSCC xenograft model

To determine whether clotrimazole affects the apoptosis of tumor cells in vivo, we further analyzed the apoptotic tumor cells and the expressions of Bcl-2 and Bax in xenograft tumors by Immunohistochemistry and western blot analysis, respectively. Our results showed that clotrimazole increased the number of apoptotic tumor cells in treated mice tissue compared with control mice (Fig. 6E). Moreover, clotrimazole treatment induced a 1.5-fold decrease of Bcl2 expression and 2.0-fold increase of Bax level compared to the control mice (Fig.5). These results indicated that, similar to the results in vitro, clotrimazole decreased tumor growth in vivo through the induction of cell apoptosis.

## Discussion

Clotrimazole is an important imidazole-derived antimycotic agent that is clinically safe and readily tolerated by human. Recently, clotrimazole was found to possess antitumor activity against several types of cancer cells in vitro [4], [9], [18], [19]. However, the histological types of these solid tumors that have been investigated for clotrimazole were all adenocarcinomas. There is little report about the anticancer effects of clotrimazole on SCC, which is one of the largest subset of cancer. Adenocarcinoma and SCC can show tremendous differences in their presenting symptoms, natural histories, prognosis, and responses to therapy [20]. Hence, we selected oral squamous cell carcinoma as the representative one of SCC to assess the anticancer effect of clotrimazole. OSCC is the most common type of head and neck squamous cell carcinoma, which is the sixth most common cancer worldwide [21]. Despite evolution in management, the prognosis and survival of OSCC patients is still poor. Thus, the identification of novel and effective therapeutic agents to inhibit OSCC cell growth is essential. In the current study, we demonstrated that clotrimazole exerted potent anticancer effect on human OSCC cells both in vitro and in vivo. Moreover, the inhibitory action of clotrimazole was correlated with the induction of cell cycle arrest and apoptosis in OSCC.

Published data have shown that clotrimazole reduces the viability of several types of cancer cells [3], [5], [6]. However, there is little evidence for the effect of clotrimazole on human OSCC cells. Thus, in the initial vitro study, we measured the growth inhibitory effect of clotrimazole in OSCC cells. We found that clotrimazole decreased cells viability and reduced colony formation of OSCC cells in a dose-dependent manner. The effective doses of clotrimazole (30 µM and 40 µM) used in our in vitro study were similar to those used in prior studies on breast cancer, endometrial cancer, and glioblastoma [4], [6], [12]. Our subsequent in vivo experiment further revealed that clotrimazole markedly inhibited cell proliferation and slowed the tumor growth of OSCC. Khalid et al reported that clotrimazole treatment caused a significant inhibition of intracranial tumor growth in rat vivo model of glioma [7]. Taken together, our experimental data show that clotrimazole can exert significant growth inhibitory effects on human OSCC cells, similar to the effects reported in human adenocarcinoma cells.

In order to mechanistically explain our observations, we investigated the effect of clotrimazole treatment on cell cycle and apoptosis. We found that clotrimazole blocked OSCC cycle progression at the G_0_/G_1_ phase in vitro experiment. This result is consistent with previous studies, such as glioblastoma and lung cancer [10], [22]. Liu et al observed that clotrimazole arrested cell cycle at G_0_/G_1_ phase correlating with the overexpression of p27^Kip^ and the decrease of cyclin D1 [12]. Furthermore, our results demonstrated that clotrimazole significantly induced apoptosis in OSCC cell lines which was further proven by our in vivo experiment. Several previous in vitro investigations indicated clotrimazole induced cell apoptosis in other types of cancer cells. Khalid and colleagues observed that clotrimazole sensitized cells to cisplatin by induction of apoptosis in human glioblastoma cell [10]. Meira et al. displayed the marked nuclear condensation and f-actin depolymerization after clotrimazole treatment in MCF-7 breast cancer cells [18]. Ito et al. demonstrated that clotrimazole induced depletion of intracellular Ca2+ stores and led to cell apoptosis in acute lymphoblastic leukemia cells [11]. Obviously, clotrimazole can inhibit the growth of OSCC in vitro and in vivo by cell cycle arrest and induction of cellular apoptosis. Our results disclosed that the clotrimazole-induced apoptosis parallel to the significant up-regulation of the pro-apoptotic protein Bax, the down-regulation of the anti-apoptotic protein Bcl-2, and activation of caspase-3. It is well known that the Bcl-2 family members play a central role in regulating apoptosis and dictating cell fate through an accurate balance between pro-apoptotic (Bax, Bak, and BH3-only proteins) and anti-apoptotic (Bcl-2 and its closest homologues) factors [23]. When the ratio of Bax to Bcl-2 increases, the mitochondrial permeability transition pore opens and in turn results in releasing apoptogenic mitochondrial proteins to activate caspases and induce cell apoptosis [24]. However, several previous studies have not connected the anticancer effects of clotrimazole with this apoptotic pathway. For example, the in vitro study by Horng et al. showed that clotrimazole significantly induced Ca^2+^ release from endoplasmic reticulum and also triggered apoptosis in human hepatoma cells in Ca^2+^-independent manner [25]. Therefore, to the best of our knowledge, it is the first time to report the involvement of Bcl2 family in clotrimazole-mediated apoptosis of SCC cells. Certainly, a lot of work is needed to confirm the activation of the apoptotic pathway which is regulated by BCL2 family of proteins as the universal mechanism of inhibitory action of clotrimazole in our further study.

In prior in vivo studies, the dosages of clotrimazole were ranged from 100 to 160 mg/kg/day for various time periods. In most of these studies, there was no major adverse reaction or significant change in blood or hepatic parameters [26]–[28]. Khalid et al. demonstrated that 125 mg/kg/day clotrimazole administered by intraperitoneal injection significantly inhibited intracranial glioma tumor formation and prolonged rat survival without sign or symptom of drug toxicity [7]. The doses of 150 mg/kg clotrimazole used in our study were similar to these reported in previous literature. We found that 150 mg/kg clotrimazole significantly inhibited OSCC tumor growth without any macroscopic evidence of abnormal changes in the heart, liver, spleen, kidney, stomach, and intestine. This finding was of particular importance as it was the first time that clotrimazole was shown to inhibit OSCC tumor growth in vivo. Moreover, these results provide a foundation for our further animal model experiments that utilizes clotrimazole to improve treatment outcomes in OSCC, such as the 4-nitroquinoline 1-oxide (4NQO)-induced mice tongue carcinogenesis model, which closely mimics multistage carcinogenesis of human OSCC on morphological and biological concepts [29].

## Conclusions

In summary, our results revealed that clotrimazole inhibited OSCC cell proliferation and tumor growth in vitro and in vivo, possibly by suppressing the apoptosis-related molecules. These results suggest that clotrimazole could be a potential therapeutic agent to block the progression of OSCC. Our results provide evidence for future clinical strategies that utilize clotrimazole as a single agent or adjuvant chemotherapeutic reagents to improve treatment outcomes in OSCC.

## Supporting Information

Figure S1 **Clotrimazole treatment does not affect body weight of OSCC xenografted nude mice.** A total of 5×10^6^ CAL27 cells/mouse were injected subcutaneously into the back next to the right front limb. When a tumor became palpable, clotrimazole (150 mg/kg/body) was administered intraperitoneally for 2 weeks, 6 times per week, control mice treated with equal volume of peanut oil. The body weight of xenografted nude mice (n = 12) was measured every two days for Fourteen days, and means of the body weight of each group were presented by mean ± (SD). Statistical significance was determined by Student t-test when compared with the control. *P*>0.05.
(TIF)

Figure S2 **The gross observation of the organs and histopathology of OSCC xenograft.** (A) The gross observation of the heart, liver, spleen, kidney, and gastrointestinal tract in control and clotrimazole-treated mice. (B) The histopathology of OSCC xenograft stained by hematoxylin and eosin in control and clotrimazole-treated mice. Original magnifications, ×200. Bar: 50 µM.
(TIF)
