# Expression of Concern: The *in vitro* and *in vivo* antitumor effects of clotrimazole on oral squamous cell carcinoma

**DOI:** 10.1371/journal.pone.0231686

**Published:** 2020-04-14

**Authors:** The PLOS ONE Editors

After publication of this article [1], concerns were raised regarding duplication of well images within Fig 2, and the representation of western blot loading controls in Fig 5. Specifically,

- In Fig 2A, the CAL27 0µM well image is incorrect, and shows a duplicate of the SCC25 0µM well image
- In Fig 2A, the SCC25 30µM well image is incorrect and shows a duplicate of the CAL27 30µM well image.
- Fig 5A and 5B incorrectly show a shared a-tubulin loading control for Bax and Bcl-2 for each experiment. Samples were run on separate gels for Bax and Bcl-2, but the loading controls for the Bcl-2 blots are not shown.

The authors have stated there were errors in the selection of images for the published Fig 2A. Further explanation of the methodology is provided by the authors as follows:

In the cell colony formation assay, the OSCC cells were seeded into 6-well plates and treated with different concentrations of clotrimazole after 24 h. At the end of the experiment, the colonies consisting of 50 cells were counted under a microscope. All experiments were repeated in triplicate. Images were captured of each well in two of the experiments.

Editorial assessment of underlying images for available replicates identified further similarities between published well images and replicate well images labelled as different cell lines. As such, concerns about the accuracy of the image records cannot be fully resolved. The quantitative data underlying the charts in Fig 2B is provided here as S1 File.

The available underlying western blot images for Fig 5 are provided in S2 File, including the a-tubulin loading control for the Bcl-2 blot for Fig 5A UM1 cells. The corresponding a-tubulin loading controls for the Bcl-2 blots for Fig 5A CAL27 cells and for Fig 5B are not available. Additionally, the underlying Bax western blots for Fig 5A are not available. Some experimental replicate blots and their associated quantification data are also provided in S2 File. The underlying data for the other figures in the article are not available.

The *PLOS ONE* Editors issue this Expression of Concern to alert readers to the concerns about Fig 2A, and the inaccurate representation of the western blots in Fig 5.

## Supporting information

S1 File Fig 2B chart data. (XLSX)

S2 File Fig 5 Underlying blot data for the representative blots and replicate blot data. (ZIP)

## References

[1] Wang J, Jia L, Kuang Z, Wu T, Hong Y, Chen X, et al (2014) The *In Vitro* and *In Vivo* Antitumor Effects of Clotrimazole on Oral Squamous Cell Carcinoma. PLoS ONE 9(6): e98885 10.1371/journal.pone.0098885 24892421 PMC4043897

