# Supplementary figures and images for: The In Vitro and In Vivo Antitumor Effects of Clotrimazole on Oral Squamous Cell Carcinoma
Source: PLoS One. 2014 Jun 3;9(6):e98885. doi: 10.1371/journal.pone.0098885 (PMC4043897; doi:10.1371/journal.pone.0098885)

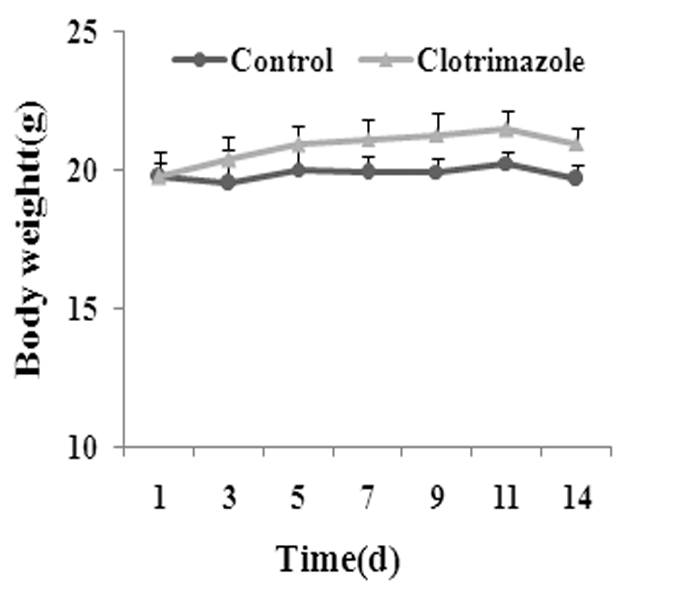

Supplement: Figure S1 — Clotrimazole treatment does not affect body weight of OSCC xenografted nude mice. A total of 5×10^6^ CAL27 cells/mouse were injected subcutaneously into the back next to the right front limb. When a tumor became palpable, clotrimazole (150 mg/kg/body) was administered intraperitoneally for 2 weeks, 6 times per week, control mice treated with equal volume of peanut oil. The body weight of xenografted nude mice (n = 12) was measured every two days for Fourteen days, and means of the body weight of each group were presented by mean ± (SD). Statistical significance was determined by Student t-test when compared with the control. P>0.05. (TIF) [file pone.0098885.s001.tif]

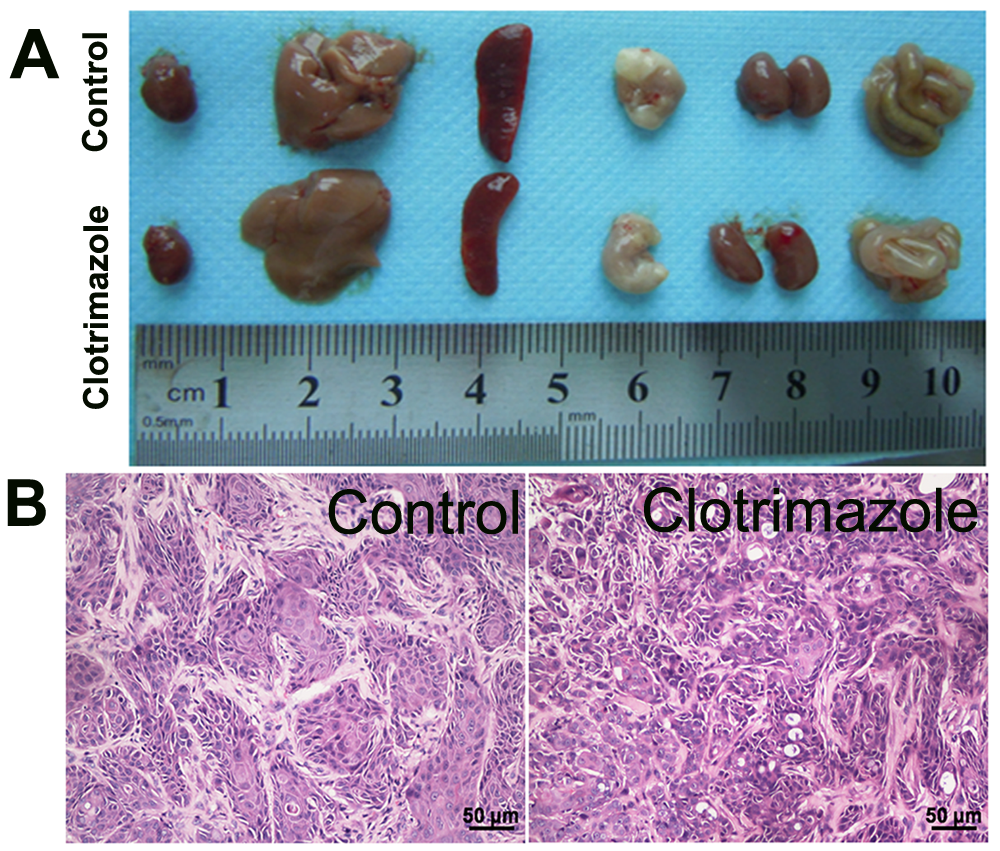

Supplement: Figure S2 — The gross observation of the organs and histopathology of OSCC xenograft. (A) The gross observation of the heart, liver, spleen, kidney, and gastrointestinal tract in control and clotrimazole-treated mice. (B) The histopathology of OSCC xenograft stained by hematoxylin and eosin in control and clotrimazole-treated mice. Original magnifications, ×200. Bar: 50 µM. (TIF) [file pone.0098885.s002.tif]
